# Crimean-Congo hemorrhagic fever viral RNA-free hard ticks infesting livestock in a rural region of southern Iran

**DOI:** 10.1016/j.parepi.2026.e00518

**Published:** 2026-05-28

**Authors:** Parisa Moazzeni, Mohammad Djaefar Moemenbellah-Fard, Hamzeh Alipour, Ali Dianat, Marzieh Shahriari-Namadi

**Affiliations:** a Student Research Committee, Department of Biology and Control of Disease Vectors, School of Health, Shiraz University of Medical Sciences, Shiraz, Iran; b Research Center for Health Sciences, Institute of Health, Department of Biology and Control of Disease Vectors, School of Health, Shiraz University of Medical Sciences, Shiraz, Iran

**Keywords:** Viral fever, Tick-borne disease, Virus, Iran

## Abstract

Tick-borne viral diseases, including Crimean-Congo hemorrhagic fever (CCHF), are often fatal infectious diseases if left untreated. They impose a significant burden of morbidity on human health. Given the history of this disease in Fars province, Iran, and the CDC report on a recent fatal case in a remote county, and since the infectivity of ticks in this area has so far been neglected, this study was undertaken to investigate the above issue. Ticks were collected from seven stations. Morphological identification was conducted using valid taxonomic keys. Molecular tests were implemented on them. From a total 361 collected hard ticks, four species across two genera of *Hyalomma* (71%) and *Rhipicephalus* (29%) were identified. Significant differences in the frequency of *Hyalomma* genus with different vertebrate hosts in this area were outlined. The implementation of RT-PCR test on all samples was negative, and the expected band was observed in none of them. To ensure the correctitude of infection test negativity, nested-PCR was performed on all RT-PCR products using the right primers. All samples exhibited no trace of infection with CCHFV genomic remnants too.

## 1 Introduction

The global dispersion of obligatory blood-feeding ticks and various tick-borne viruses (TBVs) unveils the possibility of tick-transmitted infection emergence (Shahhosseini et al., 2021). Ticks infest an extensive variety of vertebrates from lizards and tortoises to domestic animals and humans (Adeli-Sardou et al., 2019). There are currently 50 different species across 11 genera of ticks in this country (Tavassoli et al., 2024).

From a recent meta-analysis review on tick-borne pathogens in Iran, some 28 tick species across nine genera; including *Alveonasus*, *Argas*, *Boophilus*, *Dermacentor*, *Haemaphysalis*, *Hyalomma*, *Ixodes*, *Ornithodoros*, and *Rhipicephalus;* have been shown to be infected with at least 20 pathogens within 10 microbial genera; comprising *Aegyptianella*, *Anaplasma*, *Babesia*, *Borrelia*, *Brucella*, *Orthonairovirus* (*e.g.* Crimean-Congo hemorrhagic fever virus, CCHFV), *Coxiella*, *Ehrlichia*, *Rickettsia*, and *Theilleria;* from 26 Iranian provinces (Khoobdel et al., 2021).

After West Nile Virus fever, the second most prevalent worldwide arbovirus disease is CCHF (Kassiri et al., 2020). It is endemic in Asia (Afghanistan, China, India, Iran, Iraq, Kazakhstan, Kuwait, Oman, Pakistan, Saudi Arabia, Tajikistan, United Arab Emirates, Uzbekistan), Africa (Egypt, Mauritius, South Africa), and Europe (Gholizadeh et al., 2022). It was first identified in the Crimean Island of Russia in 1944, and subsequently in 1956 in the Democratic Republic of Congo (Bente et al., 2013). Its present mortality rate among humans is about 10–40% (Sadeghi et al., 2023). A pathogenic *Orthonairovirus* (Family: Nairoviridae; Order: Bunyavirales) causing this disease is transmitted by hard ticks and soft ticks to human and his domesticated livestock (Hanafi-Bojd et al., 2021).

The first cases of the disease in Iran were observed in the 1970s with the detection of CCHF antibodies in human and livestock serum. (Chinikar et al., 2009) The CCHF in Iran is primarily associated with two tick species, including *Hyalomma marginatum* and *Hyalomma anatolicum*, being particularly prevalent in the central region. Interestingly, aside from these primary vectors, there are other hard tick species, such as *Hyalomma dromedarii*, *Hyalomma detritum*, and *Hyalomma asiaticum* that have been identified as positives for the virus genome (Tavassoli et al., 2024).

The risk factors associated with this disease include contact with infected tissue and blood of patient or livestock during acute viral stage, infected tick bite and/or bare handling of tick (Aslam et al., 2023). The clinical symptoms of this disease could be headache, high fever, body pain, vomiting, and more severe signs of extensive hemorrhage with subdermal petechiae, shock, diffuse intravascular coagulation (DIC), and multi-organ deficiencies, which may lead to death. In addition, rare symptoms like intracerebral hemorrhage, compartment syndrome, intra-visceral pleural and pericardial effusions, acute pancreatitis, myocarditis, and cholecystitis has been reported (Kouhpayeh, 2019).

The CCHFV diagnosis could be performed with enzyme-linked immunosorbent assay (ELISA) alongside other laboratory tests. Except the anti-tick (*Boophilus microplus*, a one-host tick) vaccine of Bm86, no successful CCHFV vaccines has so far been available. Research trials are, however, currently in progress in the molecular entomology laboratory of Shiraz School of Health, Shiraz, Iran, to produce a virus RNA vaccine impeding the prolific distribution of CCHFV (Gholizadeh et al., 2022; Shahriari-Namadi et al., 2025).

The keystone treatment of CCHF is supportive therapy. Despite ribavirin administration as specific anti-viral drug treatment since years ago, its clinical effectiveness is still disputable, which needs more randomized controlled clinical trials (RCTs) to verify and recommend it for CCHF treatment. Furthermore, other therapeutic strategies; such as steroids, immunoglobulins, and monoclonal antibodies (Mo-Abs) administration; require more definitive data. At present, a promising antiviral drug to cure CCHF is favipiravir (Kouhpayeh, 2021).

Iran is known to be endemic for CCHF. According to the latest meta-analysis study, the cumulative prevalence of CCHFV has been 6% (CI: 4.5–7.9%, 95%), and for regions above 1001–1500 m a. s. l. 4.6% (CI: 4.3–9.5%, 95%) (Sadeghi et al., 2023). Most human cases have been reported from Sistan-Baluchistan, Isfahan, Fars, Khorasan, and Khuzestan provinces (Farhadpour et al., 2015). Ticks like *Hy. marginatum, Hy. anatolicum, Hy. asiaticum, Hy. dromedarii, Rhipicephalus sanguineus,* and *Rhipicephalus appendiculatus* are known to be the most prevalent CCHFV-positive species (Telmadarraiy et al., 2015; Farhadpour et al., 2016; Fakoorziba et al., 2015; Fakoorziba et al., 2012).

In an epidemiologic study of CCHF in the southwest province of Khuzestan from 1999 to 2015, a total of 82 probable cases were reported, 18 of whom culminated in death. From 31 confirmed human patients, 9 deaths were reported. Males contributed to most cases. The highest occupational risk groups were farmers, butchers, and slaughter house workers. Most cases of disease were reported in spring and summer when the ticks are highly active. (Sharififard et al., 2016)

Considering the history of this disease in Fars province, and according to the CDC report from Shiraz University of Medical Sciences (SUMS) on the infection of individuals with CCHF alongside a fatal case in Zarrindasht county during 2022, and since studies on the infectivity of ticks in this county has so far been neglected, this study was undertaken to investigate the above issue.

## 2 Materials and methods

### 2.1 Study area

**Fig. 1:**
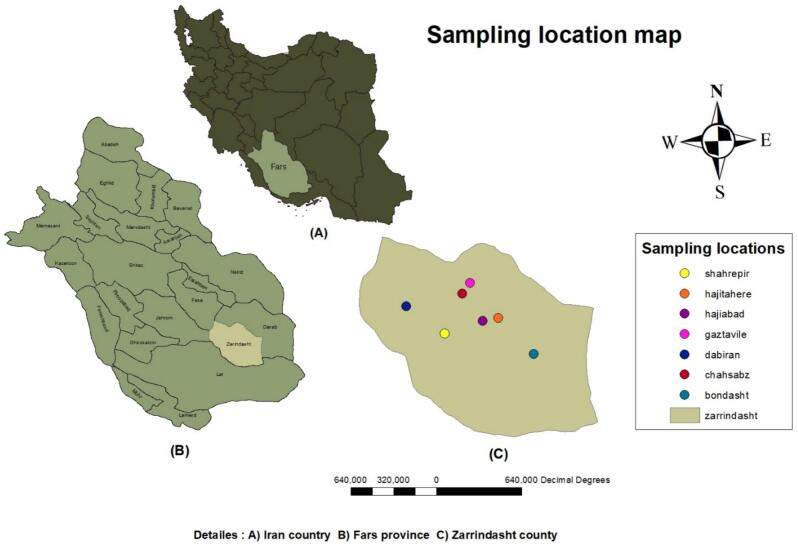
The sampling location map of country Iran (A), Fars province (B), and Zarrindasht county encompassing the seven sampling stations.

Fig. 1 illustrates the sampling locations map in the rural county of Zarrindasht, Fars province, Iran. It lies on the southeast of this province with the main township of Hajiabad extending on the coordinates of 54^°^25′E and 28^°^22′N, at an altitude of 870 m above sea level. Its mean annual temperature and mean annual precipitation approximates to 22 °C and 170 mm, respectively. Agricultural activities are the main occupation of its residents. Livestock farming on sheep, goats, cows, camels and other domesticated animals is the routine way of life for some people in this area.

### 2.2 Sampling and identification of ticks

The tick samples were collected from seven stations of this county in Hajiabad, Shahrepir, Dabiran, Hajitahere, Geztevileh, Bondasht and Chahsabz (Fig. 1). Ticks were manually picked from their hosts including sheep, goats and cattle as well as dogs using finely curved-tip forceps under standard health protocols and transferred to sterile vials. Data on type of host, date and place of samplings, coordinates of sample sites were recorded. Live ticks were then transferred to the entomology lab and directly maintained in a freezer at −70 °C. Morphological identification of ticks was conducted under ice cold chain conditions using valid taxonomic keys as well as a Zeiss compound binocular microscope (Hosseini-Chegeni, 2021; Estrada-Peña et al., 2004).

### 2.3 Primer design

Primers were designed from the S segment gene region to assert the positivity of CCHFV genome. To design primers, the S segment sequences of this virus previously registered in GenBank by other researchers were extracted and aligned using the MEGA6.0 software. From the conserved regions, two pairs of primers were then utilized. Table 1 illustrates the primers used to trace CCHFV genomic relics in this study.

**Table 1:** Characteristics of designed primers.

No.	PCR Method	Primer sequence	Characteristics
1	RT-PCR	Forward: ACCTATATACGAATGTGCCTGG	Tm 54 °C, Loop = 0, 22 bp
2	RT-PCR	Reverse: ACAACATCTCTTTGACAGACATTAC	Tm 54 °C, Loop = 0, 24 bp
3	nested RT-PCR	Forward: AGAAGGGACTGGAGTGGTTC	Tm 55 °C, Loop = 0, 19 bp
4	nested RT-PCR	Reverse: GCATTGACACGGAAGCCTATG	Tm 55 °C, Loop = 0, 19 bp

### 2.4 Extraction of RNA and molecular diagnosis

Ticks were separated on the basis of the species and place of sampling, and they were pooled using liquid nitrogen. In general, in each case, 5 tick specimens representing a specific tick species from a particular locality were pooled together, and RNA extraction was performed. Their RNAs were then extracted according to the specific kit guidelines from Sambio company (SAMBIO, Taiwan).

### 2.5 cDNA synthesis

Following RNA extraction, cDNA synthesis was immediately performed using Sambio Co. specific kit and strictly pursuing the kit protocol. The cDNA was synthesized in a total volume of 20 μl, including 10 μl kit specific buffer, 2 μl Reverse Transcriptase enzyme, 1 μl RNA, and 7 μl ddH_2_O for 10s at 25 °C, 60 min at 47 °C, and 5 min at 85 °C in a thermocycler device.

### 2.6 RT-PCR

The gene region considered to investigate the infectivity was the S segment of the CCHFV genome. Molecular investigation was performed in two steps: The first step included reverse transcription polymerase chain reaction (RT-PCR), and the second step to ensure the correctitude of results was pursued by nested RT-PCR. The RT-PCR was conducted in a total volume of 20 μl, including 8.5 μl ddH_2_O, 8.5 μl amplicon Master mix, 1 μl forward primer, 1 μl reverse primer, and 1 μl cDNA for 5 min at 94 °C, and 35 cycles of 30s each at 94 °C, 54 °C, and 72 °C, and finally 10 min at 72 °C as a final extension step. The nested RT-PCR was also implemented with the same protocol, except with an annealing temperature of 55 °C. The PCR products were visualized by electrophoresis technique in 1% agarose gel using the safe stain.

### 2.7 Positive control conduct

In order to confirm the PCR positive control sample, a pair of routine primers, GAPF and GAPR, was used to replicate the insects' Glyceraldehyde 3-phosphate dehydrogenase (GAPDH) gene. In the next step, part of the acquired cDNA was run for RT-PCR with the GAPDH primer, and GAPDH enzyme in another microtube as a positive control, to ensure the presence of GAPDH enzyme. Eventually, an expected band of 500 bp was observed, which verified the method, RNA extraction, cDNA synthesis, and also the correct PCR performance.

### 2.8 Data analysis

This study utilized Chi-square test to assess the relationship between the two qualitative variables of captured species acting commonly as vectors of CCHFV, including *Hy. marginatum* and *Hy. anatolicum*, with the hosts on whom these tick species have been engorging.

## 3 Results

**Fig. 2:**
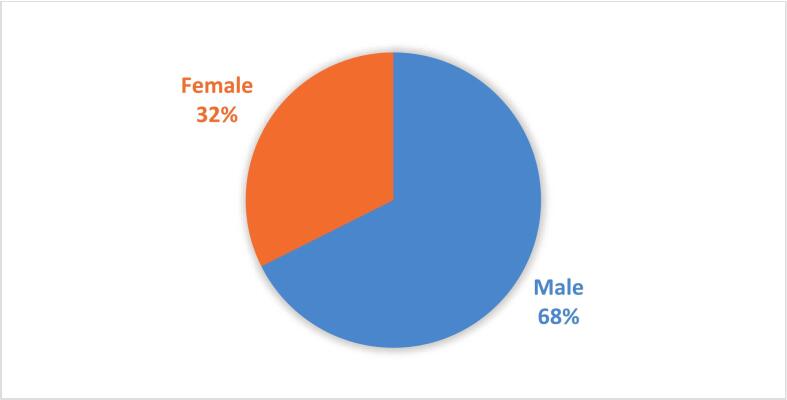
Pie chart shows the relative proportion of ticks by gender captured in Zarrindasht County.

Table 2 exhibits the percentage distribution of hard ticks on their vertebrate hosts. From a total 361 collected hard ticks, four species across two genera of *Hyalomma* (71%) and *Rhipicephalus* (29%) were identified (Fig. 2). Both the maximal (26%) and minimal (1.66%) percentage distribution of ticks belonged to *Hy. marginatum* on sheep and cow, respectively. No *Hy. anatolicum* was found on goats in this study area. Table 3 highlights the statistical test performed between the *Hyalomma* tick species frequencies on different hosts. The Chi-square test showed that there were significant statistical differences in the frequency of this tick taxon with different vertebrate hosts in the Zarindasht county (*P* < 0.05), such that most *Hyalomma* tick species (70%) were associated with sheep hosts.

**Table 2:** Distribution of *Hyalomma* and *Rhipicephalus* tick species on their hosts.

Host/Species	Sheep (%)	Cow (%)	Goat (%)	Dog (%)
*Hy. marginatum*	26.04	1.66	8.03	0.00
*Hy. anatolicum*	19.11	16.07	0.00	0.00
*Rh. turanicus*	3.05	0.00	0.00	0.00
*Rh. sanguineus*	0.00	0.00	0.00	26.04

**Table 3:** Chi-square analysis for the association of *Hyalomma* vector species with the host.

Test Statistics		
Parameter	Species	Host
Chi-square	0.016^a^	1.132E2^b^
Df	1	2
Asym. sig.	0.901	0.000

a. 0 cells (0.0%) have expected frequencies less than 5. The minimum expected cell frequency is 128.0.

b. 0 cells (0.0%) have expected frequencies less than 5. The minimum expected cell frequency is 85.3.

**Fig. 3:**
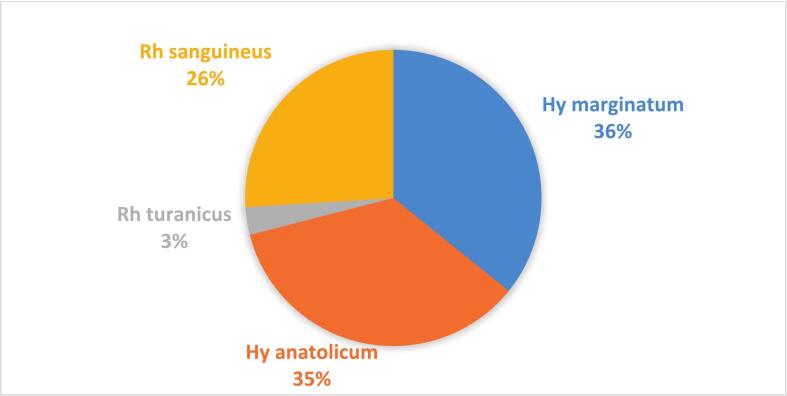
Pie chart shows the relative proportion of ticks by species captured in Zarrindasht County.

Male ticks represented a larger (67.6%) proportion than females (32.4%) in the study sample (Fig. 3). Most ticks (48.1%) were associated with sheep, while goat was the least infested host (8%) among the four categories of livestock. The most abundant (35.7%) tick species was *Hyalomma marginatum*, while *Rhipicephalus turanicus* was the least (3%) frequent species among all ticks occurring only on sheep (Table 4). The brown-dog tick, *Rhipicephalus sanguineus*, was also found specifically on dogs (26%).

**Table 4:** Characteristics of collected ticks.

Species	Gender (%)		Month (%)				Total (%)
	Male	Female	October	November	February	June	
*Hy. marginatum*	92 (25.5)	37 (10.2)	45 (12.5)	30 (8.3)	46 (12.7)	8 (2.2)	129 (35.7)
*Hy. anatolicum*	64 (17.7)	63 (17.5)	70 (19.4)	4 (1.1)	27 (7.5)	26 (7.2)	127 (35.2)
*Rh. turanicus*	–	11 (3)	11 (3)	–	–	–	11 (3)
*Rh. sanguineus*	88 (24.4)	6 (1.7)	–	–	–	94 (26)	94 (26)
Total	244 (67.6)	117 (32.4)	126 (34.9)	34 (9.4)	73 (20.2)	128 (35.4)	361

**Fig. 4:**
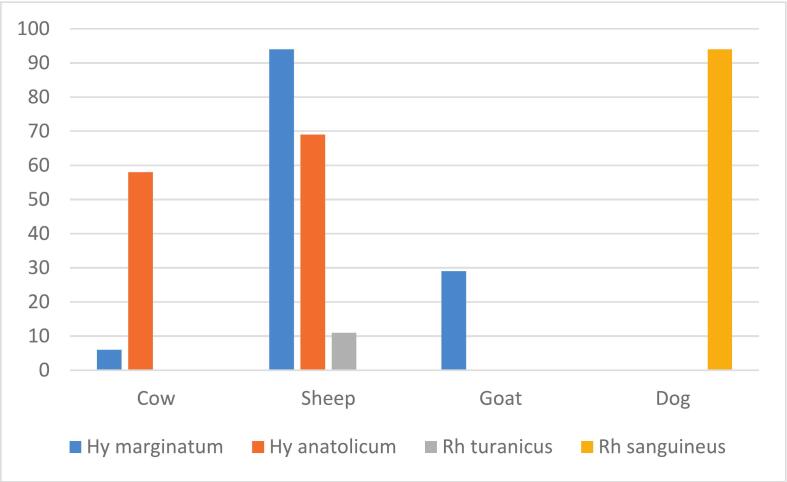
Tick capture results in Zarrindasht County by host.

Sheep were mostly infested with *Hy. marginatum* (26%) and *Hy. anatolicum* (19%), but least infested with *Rh. turanicus* (3%). In contrast, cattle were mostly infested with *Hy. anatolicum* (16%), but least infested with *Hy. marginatum* (1.7%). All *Rh. sanguineus, Hy. marginatum*, and *Rh. turanicus* ticks were solely removed from dog (26%), goat (8%), and sheep (3%), respectively (Fig. 4).

These ticks were sampled in October (35%), November (9.4%), February (20.2%) and June (35.4%) of 2023–2024. The ‘brown-dog tick’, *Rh. sanguineus*, was the most frequently (26%) collected species of all samples in June, while *Hy. anatolicum* was the least frequently (1.1%) collected species in November. In addition, most ticks (44.3%) were found in the Hajiabad sampling site, while the lowest abundance (13.5%) of ticks was observed in Shahrepir location.

The implementation of RT-PCR test on all samples was negative, and the expected band was observed in none of them. At the next stage to ensure the correctitude of infection test negativity, nested-PCR was performed on all RT-PCR products using the right primers. All samples exhibited no trace of infection with CCHFV genomic remnants too.

## 4 Discussion

In the present research, hard tick samples from seven different stations in Zarindasht county, Fars province of southern Iran, were screened for possible infection with Crimean-Congo Hemorrhagic Fever Virus (CCHFV). The genomic remnants of this virus were detected in none of the collected samples. Despite the fact that the southern parts of this province including Zarindasht county are endemic foci of CCHF in Iran, and the previous reports of settlers' affliction with this disease, given a recent human death case from this county, as well as strict adherence of all stages to the national reference lab protocols, the current data reflected the absence of viral genomic remnants in the collected hard ticks.

The significance of the present findings lies in the strong virulence, requiring a Biosafety Level IV in clinical follow-ups, and possible sudden rise of viral genomic remnants during specific periods in infected ticks. This does not, however, reflect that the virus is absent from these settings. Among the possible factors of influencing the fine detection of viral genome, season of tick sampling, climate change, cold-chain preservation and handlings, and other pitfalls could be responsible for this outcome.

The reasons for clustered sampling of ticks in the above-named periods were the disappearance of ticks in cold winter months, the short time-interval allocated to this investigation due to academic limitations, and the pre-spraying with insecticides at some stations. Further research is needed to assert the hard tick species diversity and population structure in Fars Province, and to determine their infection with CCHFV at different intervals of time using specific PCR methods.

## 5 Conclusion

It was concluded that, despite the common presence of CCHFV vectors in this area, no traces of viral RNA genomic remnants indicated that this virus activity could be sporadically discerned in the study area.

## CRediT authorship contribution statement

**Parisa Moazzeni:** Writing – original draft, Software, Methodology, Investigation, Data curation. **Mohammad Djaefar Moemenbellah-Fard:** Writing – review & editing, Validation, Supervision, Resources, Project administration, Funding acquisition, Conceptualization. **Hamzeh Alipour:** Writing – review & editing, Validation, Supervision, Software, Methodology, Investigation, Formal analysis, Conceptualization. **Ali Dianat:** Writing – original draft, Validation, Resources, Methodology, Investigation, Data curation. **Marzieh Shahriari-Namadi:** Writing – original draft, Validation, Software, Project administration, Investigation, Data curation.

## Ethics statement

All authors consented to participate in this study, which was conducted based on the Declaration of Helsinki. The proposal was approved by the Ethics Committee of Shiraz University of Medical Sciences (IR.SUMS.SCHEANUT.REC.1402.124), and the research project code was 29067. This work was reviewed, approved, and funded to the main corresponding author (M.D.M-F) by Shiraz University of Medical Sciences (SUMS), Shiraz, Iran.

## Declaration of competing interest

All authors declare that they have no known competing financial interests or personal relationships that could have appeared to influence the work reported in this paper. The authors declare no conflicts of interest.

## Data Availability

The data supporting the results of this research are all contained within the manuscript.
